# P017 Serial DXA Monitoring in Aromatase Inhibitor-Induced Bone Loss: A Decade of Progressive Bone Loss, Stabilisation with Bisphosphonate Therapy and Recovery Following Endocrine Therapy Cessation

**DOI:** 10.1093/rap/rkag113.047

**Published:** 2026-11-03

**Authors:** Bilal Hameed, Hafis Muhammed, Naveen Ravichandran, Syed Mustafa, Srinivasan Venkatachalam

**Affiliations:** The Royal Wolverhampton Trust, Wolverhampton, United Kingdom; The Royal Wolverhampton Trust, Wolverhampton, United Kingdom; The Royal Wolverhampton Trust, Wolverhampton, United Kingdom; The Royal Wolverhampton Trust, Wolverhampton, United Kingdom; The Royal Wolverhampton Trust, Wolverhampton, United Kingdom

## Abstract

**Introduction:**

Aromatase inhibitors (AIs) substantially reduce breast cancer recurrence but accelerate bone loss through profound oestrogen deprivation. International guidelines recommend fracture risk assessment, serial dual-energy X-ray absorptiometry (DXA), optimisation of calcium and vitamin D, and anti-resorptive therapy when indicated. Despite these measures, uncertainty remains regarding the optimal management of patients who continue to lose bone mineral density (BMD) during AI therapy. We present a 10-year longitudinal case illustrating how serial DXA surveillance informed management, demonstrated the natural history of AI-induced bone loss, and reinforced the importance of multidisciplinary care throughout breast cancer survivorship.

**Case description:**

A **55-year-old woman** was diagnosed with **Grade 3 ER-positive, PR-positive, HER2-negative invasive ductal breast carcinoma at 41 years of age**. Treatment included neoadjuvant chemotherapy (FEC followed by Taxotere), breast-conserving surgery, bilateral oophorectomy, tamoxifen, and **letrozole from April 2015 to June 2023**.

At commencement of letrozole, her only recognised osteoporosis risk factors were **low BMI (21 kg/m²)** and profound oestrogen deprivation following bilateral oophorectomy. BMI subsequently improved to approximately **22 kg/m²**. She had **no previous fragility fractures, rheumatoid arthritis, diabetes mellitus, smoking history, excess alcohol intake, recurrent falls, glucocorticoid exposure or family history of hip fracture**. Secondary causes of osteoporosis were repeatedly excluded whenever further BMD decline was observed.

Baseline DXA demonstrated **lumbar spine BMD 1.036 g/cm² (T-score −1.36)** and **femoral neck BMD 0.876 g/cm² (T-score −0.86).** The first surveillance DXA, performed two years after initiating letrozole in accordance with AI-induced bone loss (AIBL) guidance, demonstrated a marked **decline in lumbar spine BMD to 0.927 g/cm² (T-score −2.1),** representing an **annual loss of 4.3%,** with femoral neck BMD declining to 0.828 g/cm² (T-score −1.3). In response to this significant deterioration, **oral risedronate was commenced**, while calcium and vitamin D supplementation were continued. The patient remained fully compliant with treatment throughout follow-up.

Subsequent serial DXA demonstrated marked slowing and stabilisation of bone loss, with lumbar spine BMD measuring 0.897 g/cm² (T-score −2.4) in 2019 and 0.882 g/cm² (T-score −2.5) in 2021, while femoral neck BMD remained largely stable (0.839 and 0.843 g/cm², respectively). Following **discontinuation of letrozole in 2023, lumbar spine BMD improved to 0.918 g/cm² (T-score −2.4),** with stable femoral neck BMD (0.845 g/cm²; T-score −1.4), despite no additional osteoporosis treatment. Importantly, she remained fracture-free throughout follow-up.

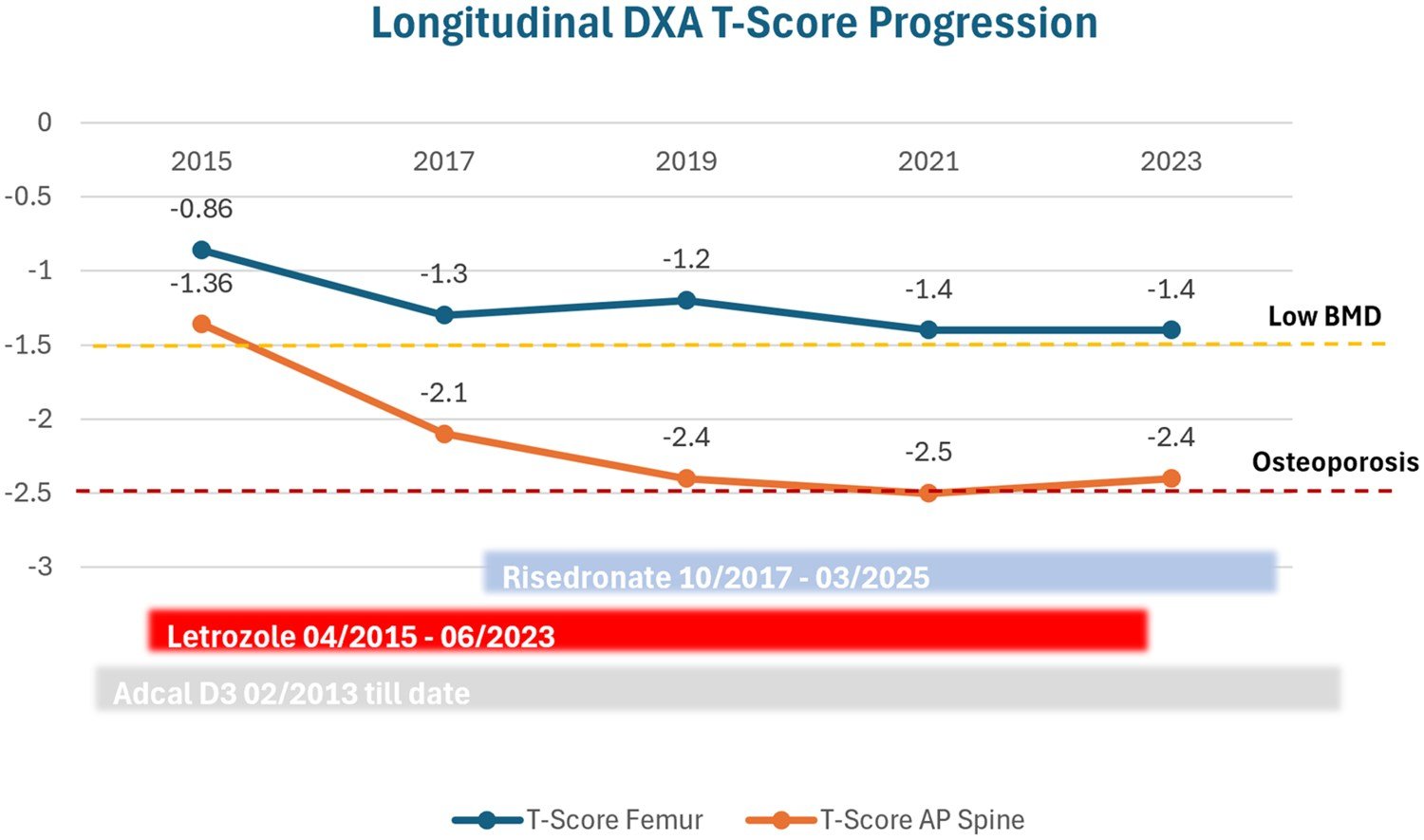

**P017 Figure 1**

**Discussion:**

This case highlights the value of **serial DXA surveillance** in understanding the trajectory of aromatase inhibitor-induced bone loss (AIBL) and supporting clinical decision-making. The most striking finding was the rapid decline in lumbar spine BMD within the first two years of letrozole therapy, despite calcium and vitamin D supplementation. This prompted initiation of oral risedronate in accordance with current AIBL guidance.

Following commencement of risedronate, serial DXA demonstrated **stabilisation of bone loss**, with a marked reduction in the rate of lumbar spine decline and preservation of femoral BMD. The patient remained fully compliant with treatment, sustained no fragility fractures, and repeated assessment excluded secondary causes of osteoporosis. These findings suggest that anti-resorptive therapy effectively attenuated further skeletal deterioration during continued aromatase inhibitor exposure.

This case also highlights the challenge of interpreting serial DXA findings during prolonged endocrine therapy. Although lumbar spine BMD remained in the osteoporotic range, the absence of fractures, stable BMD trajectory following risedronate, low overall fracture risk and anticipated completion of endocrine therapy supported continuation of guideline-based management rather than escalation to more potent anti-resorptive or anabolic therapy. The subsequent improvement in lumbar spine BMD following discontinuation of letrozole reinforces the central role of persistent oestrogen deprivation in AI-induced bone loss and suggests that recovery may not occur until endocrine therapy has been completed.

Importantly, this case reinforces the value of multidisciplinary collaboration between oncology and metabolic bone services. Regular DXA surveillance enabled timely intervention, informed shared decision-making and prevented fragility fractures throughout treatment. It also provides a practical learning point for clinicians: during ongoing aromatase inhibitor therapy, stabilisation of BMD may represent an appropriate therapeutic response, while meaningful recovery may only be expected after cessation of endocrine therapy.

**Key learning points:**

• Serial DXA monitoring provides valuable insight into the trajectory of AI-induced bone loss and informs treatment decisions.

• The greatest lumbar spine BMD decline may occur during the first two years of aromatase inhibitor therapy.

• Bisphosphonate therapy may substantially slow bone loss, even when BMD does not immediately improve.

• Stabilisation rather than recovery of BMD may represent an appropriate treatment response while profound oestrogen deprivation persists.

• Multidisciplinary management and guideline-directed surveillance enabled this patient to remain fracture-free throughout treatment.

• Improvement in BMD after AI cessation highlights the central role of oestrogen in skeletal homeostasis.

